# Prevalence and associated factors of shisha smoking among students in Senegal: Global Youth Tobacco Survey 2020

**DOI:** 10.18332/tid/186656

**Published:** 2024-05-14

**Authors:** Bai Cham, Scott R. Weaver, Candace K. Jones, Lucy Popova, Nerline Jacques

**Affiliations:** 1 School of Public Health, Georgia State University, Atlanta, United States; 2 Disease Control and Elimination Theme, Medical Research Council Unit The Gambia, London School of Hygiene and Tropical Medicine, Banjul, The Gambia; 3 Office on Smoking and Health, National Center for Chronic Disease Prevention and Health Promotion, Centers for Disease Control and Prevention, Atlanta, United States; 4 Research Triangle Institute (RTI) International, Research Triangle Park, United States; 5 CyberData Technologies, Herndon, United States

**Keywords:** tobacco, shisha smoking, Senegal, Global Youth Tobacco Survey

## Abstract

**INTRODUCTION:**

Although shisha smoking is banned in Senegal, it has become increasingly popular, especially among youth. Despite the health risks associated with shisha smoking, there are few studies on shisha smoking in West Africa and none in Senegal. Our study assessed the prevalence and factors associated with shisha smoking among students aged 13–15 years in Senegal.

**METHODS:**

We used the 2020 Global Youth Tobacco Survey (GYTS) Senegal data from 2524 students aged 13–15 years. We calculated the weighted prevalence of ever and current (past 30 days) shisha smoking. Multivariable logistic regression analyses identified factors associated with ever and current shisha smoking among students.

**RESULTS:**

The prevalences of ever and current shisha smoking were 9.8% (95% CI: 7.7–12.3) and 2.2% (95% CI: 1.5–3.1), respectively. Ever shisha smoking was significantly associated with being male (AOR=1.97; 95% CI: 1.33–2.92), current cigarette smoking (AOR=7.54; 95% CI: 2.95–19.29), higher class grade (AOR=2.27; 95% CI:1.10–4.67), more weekly pocket money (AOR=3.29; 95% CI:1.36–7.95), current use of smokeless tobacco (AOR=11.53; 95% CI: 4.98– 26.72), and exposure to secondhand cigarette smoke in public (AOR=1.55; 95% CI: 1.00–2.41). Current shisha smoking was significantly associated with current cigarette smoking (AOR=21.75; 95% CI: 6.08–77.78), more weekly pocket money (AOR=8.91; 95% CI: 1.75–45.40), current use of smokeless tobacco (AOR=8.26; 95% CI: 2.07–33.04), and fathers’ smoking (AOR=3.34; 95% CI: 1.24–8.96).

**CONCLUSIONS:**

One in 10 students aged 13–15 years have ever smoked shisha and 2.2% were currently smoking it, suggesting that shisha smoking is a public health concern in Senegal. Senegal might consider offering students more education on the harms of shisha, both in schools and through comprehensive media campaigns that address all tobacco products.

## INTRODUCTION

Shisha (also known as waterpipe, hookah, or narghile) smoking started in and was localized to the Middle East for centuries, but evidence shows it has become a global epidemic^1-5^. Many people who smoke shisha do so in groups at restaurants, cafes, bars, and other venues that promote social interaction^5^. The addition of flavors, and the social atmosphere (social interaction) associated with the way it is typically consumed, are appealing to young people^5-7^. Evidence shows that despite cigarette smoking prevalence remaining stable or declining in many countries, other forms of tobacco use, including the use of shisha, are increasingly popular especially among young people^2,4,5^.

According to some sources, shisha smoking is used with the belief that filtering tobacco smoke through water might make it less harmful^8^. This is not true, although the mistaken perception persists to this day. A report in The Gambia has shown that many people use it for fun and do not believe it has similar health risks as cigarettes^9^. In a study among high school students in Ethiopia, 38.6% of participants perceived shisha to be less harmful compared with cigarettes^10^. This misperception about its relative safety attracts many people, especially youth, to initiate smoking shisha^3^. However, evidence has shown that shisha tobacco smoke has many of the same harmful chemicals as cigarettes, including carbon monoxide, tar, and nicotine, and shisha smoking more than doubles the risk of respiratory illnesses and esophageal, oral, and lung cancer^11,12^. It is also associated with metabolic syndrome, cardiovascular diseases, and poor mental health among users, as well as low birth weight among infants of mothers who smoke shisha^12,13^.

Research has shown that in a single session, people who smoke shisha are exposed to significantly higher levels of chemicals and carcinogens compared with smoking a single cigarette^2,11^. A single shisha smoking session exposes users to 9-11 times the amount of carbon monoxide, and about twice the amount of nicotine, compared with a single cigarette^2,11,14^. The sharing of the waterpipe mouth piece is also a risk factor for the spread of infectious diseases including tuberculosis^15^.

Senegal ratified the World Health Organization (WHO) Framework Convention on Tobacco Control on 21 May 2003^16^. International treaties and national laws in Senegal protect minors (defined as children aged <18 years) from all forms of exposure to tobacco^16,17^. The 2014 Tobacco Control Act in Senegal prohibits the sale of all forms of tobacco products to minors^18^. The importation, distribution, sale, and smoking of shisha are also banned under the Act^19^. Moreover, there is a complete ban on smoking in public places, including bars and pubs^17^. The second edition of the WHO Advisory note on shisha smoking report has also shown that it is popular among young people globally^5^. Empirical research evidence is lacking, but anecdotal evidence has suggested a proliferation of bars and restaurants where shisha is advertised and served^20^. Despite the health risks associated with shisha smoking, there are few studies on shisha smoking among youth in West Africa and none in Senegal. The studies conducted in West African countries, including Ghana^21^, The Gambia^22^, Nigeria^23^ and Guinea Bissau^24^, have shown that shisha smoking is an emerging public health challenge in this region. Studies are needed to inform education campaigns, policy, and enforcement. This study assessed the prevalence and associated factors of shisha smoking among students aged 13-15 years in Senegal. In addition, we explored the main shisha smoking venues, age at first use of shisha, and average number of shisha smoking sessions in a day among students who smoked shisha.

## METHODS

### Data source

We used the Global Youth Tobacco Survey (GYTS) 2020 Senegal data. GYTS is a nationally representative school-based survey, and the sample is drawn using a two-stage cluster sampling design^25^. Schools are selected by probability proportional to size during the first stage, and then classes within participating schools are selected as a probability sampling in the second stage^26^. The GYTS 2020 in Senegal covered a sample of 4320 youths, aged 11–17+ years, with a response rate of 93.9%. We restricted our analysis to this age cohort (n=2524) because GYTS is designed to be representative of the national population of students aged 13–15 years.

### Outcomes

Ever use of shisha was measured using the question: ‘Have you ever tried or experimented with shisha smoking, even one or two puffs?’ with response options of ‘yes’ and ‘no’.

Current (past 30 days) smoking of shisha was measured using the question: ‘During the past 30 days, on how many days did you smoke shisha?’. Those who reported to have smoked one or more days in the past 30 days were defined as students who currently smoke shisha and the variable was dichotomized.

In addition, we also examined the age at first use of shisha (‘How old were you when you first tried smoking shisha?’) and the shisha smoking venues used by youth, measured by asking: ‘The last time you smoked shisha during the past 30 days, where did you smoke it?’ with response options: ‘I did not smoke shisha during the past 30 days’, ‘at home’, ‘at a coffee shop’, ‘at a restaurant’, ‘at a bar or club’, and ‘other’. We explored the average number of shisha smoking sessions in a day by asking: ‘Please think about the days you smoked shisha during the past 30 days. How many shisha smoking sessions did you usually participate in per day?’. We also assessed if students who currently smoked shisha were refused to be served shisha in the past 30 days because of their age, by asking: ‘During the past 30 days, did anyone refuse to serve you shisha because of your age?’.

### Exposure variables

Our exposure variables included sex, class grade, average weekly pocket money, parents’ working status, cigarette smoking status, current use of smokeless tobacco, parents’ smoking status, closest friends’ smoking status, and secondhand smoke exposure (home and public). The classifications for all of these variables are given in Table 1. We used current cigarette smoking and ever tried cigarette smoking variables to create the cigarette smoking status variable. Current cigarette smoking is defined in GYTS as having smoked cigarettes within the past 30 days using the question: ‘During the past 30 days, on how many days did you smoke cigarette?’^27^. Ever tried smoking cigarettes was defined by asking: ‘Have you ever tried or experimented with cigarette smoking, even one or two puffs?’^27^. Cigarette smoking status was classified into never smoked cigarettes, ever tried cigarette smoking but not currently smoking, and currently smoking cigarettes.

**Table 1:** Characteristics of Senegalese student participants, Global Youth Tobacco Survey 2020 (N=2524)

*Characteristics*	*Unweighted frequency*	*Weighted percent (95% CI)*
**Sex**		
Boys	1111	45.7 (40.9–50.5)
Girls	1389	54.3 (49.5–59.1)
**Class grade**		
6éme (grade 7)	719	31.9 (23.6–41.6)
5éme (grade 8)	1006	33.0 (22.4–45.7)
4éme (grade 9)	549	25.4 (16.2–37.5)
3éme (grade 10)	232	9.7 (5.8–15.7)
**Average weekly pocket money** (CFA francs)		
Usually don’t have any spending money	781	34.5 (29.0–40.4)
<2500	1304	50.4 (44.5–56.2)
2500–5000	284	10.4 (7.6–14.1)
>5000	125	4.7 (3.2–6.9)
**Parents’ working status**		
Father, stepfather, or mother’s partner only	728	30.3 (27.2–33.7)
Mother, stepmother, or father’s partner only	209	8.8 (7.6–10.2)
Both	1357	52.0 (47.9–56.2)
Neither	137	6.2 (5.0–7.7)
Don’t know	58	2.6 (1.8–3.6)
**Cigarette smoking status**		
Never smoked cigarettes	2180	87.7 (85.2–89.9)
Ever tried cigarette smoking but does not currently smoke cigarettes	213	8.9 (7.1–11.2)
**Currently smoking cigarettes**	76	3.3 (2.4–4.5)
Current use of smokeless tobacco		
Yes	72	3.5 (2.2–5.6)
No	2337	96.5 (94.5–97.8)
**Exposure to SHS in public**		
Yes	974	36.4 (29.6–43.8)
No	1464	63.6 (56.2–70.4)
**Exposure to SHS at home**		
Yes	310	13.0 (9.2–18.3)
No	2069	87.0 (81.7–90.6)
**Parents’ smoking status**		
None	1919	80.2 (77.9–82.3)
Both	153	6.1 (4.8–7.7)
Father only	184	6.9 (5.7–8.4)
Mother only	12	0.4 (0.2–0.8)
Don’t know	142	6.3 (5.0–8.0)
**Closest friends’ smoking status**		
None	2192	90.5 (88.4–92.1)
Some	177	6.8 (5.5–8.3)
Most	62	2.3 (1.6–3.2)
All	12	0.5 (0.3–0.9)
**Ever use of shisha**		
Yes	290	9.8 (7.7–12.3)
No	2127	90.2 (87.7–92.3)
**Current use of shisha**		
Yes	65	2.2 (1.5–3.1)
No	2293	97.8 (96.9–98.5)

CFA Francs: West African Francs (official currency of eight countries in West Africa); 1000 CFA francs about US$1.6. SHS: secondhand smoke.

### Statistical analysis

We used Stata version 17 to conduct our data analysis^28^. All the analyses accounted for the complex survey design by incorporating sampling weights and the stratification and cluster variables using the svy command in Stata. We calculated the weighted prevalence (with 95% confidence intervals) of ever and current smoking of shisha. We conducted multivariable logistic regression analyses to identify factors associated with ever and current shisha smoking among students. The multivariable logistic regression was built based on findings from previous literature^10,21^, and also in consultation with experts in the field. All the exposure variables (covariates) mentioned above were included in the model. Age was not included in the multivariable regression model because of collinearity with class grade. We conducted chi-squared tests to compare the prevalence of shisha smoking across groups.

## RESULTS

Table 1 shows the characteristics of study participants. More than half of the students were girls (54.3%; 95% CI: 49.5–59.1). About one-third (31.9%; 95% CI: 23.6–41.6) of the students were in grade seven, one-third (33.0%; 95% CI: 22.4–45.7) in grade eight, 25.4% (95% CI: 16.2–37.5) in grade nine, and 9.7% (95% CI: 5.8–15.7) in the tenth grade. Half (50.4%; 95% CI: 44.5–56.2) said their weekly pocket money was <2500 CFA francs (about US$4), and about one-third of students indicated they usually do not have any weekly pocket money (34.5%; 95% CI: 29.0–40.4). The prevalence of current cigarette smoking was 3.3% (95% CI: 2.4–4.5), and 8.9% (95% CI: 7.1–11.2) have ever tried but were not currently smoking cigarettes. More than three-quarters (80.2%; 95% CI: 77.9–82.3) of the students indicated neither of their parents smoked tobacco. The prevalence of exposure to secondhand smoke at home was 13.0% (95% CI: 9.2–18.3), and 36.4% (95% CI: 29.6–43.8) were exposed to secondhand smoke in public places.

### Prevalence and factors associated with ever and current shisha smoking

The prevalence of ever and current shisha smoking were 9.8% (95% CI: 7.7–12.3) and 2.2% (95% CI: 1.5–3.1), respectively (Table 1). The prevalence of ever used shisha was 13.2% (95% CI: 10.4–16.6) among boys and 6.9% (95% CI: 5.1–9.3) among girls. A quarter of those who ever tried, but were not currently smoking cigarettes, had ever used shisha (25.9%; 95% CI: 17.0–37.3), and more than half of those who currently smoked cigarettes had ever tried shisha (50.5%; 95% CI: 36.9–64.9). More than one-quarter of students who currently smoked cigarettes also currently smoked shisha (26.6%; 95% CI: 15.4– 42.0).

**Figure 1:**
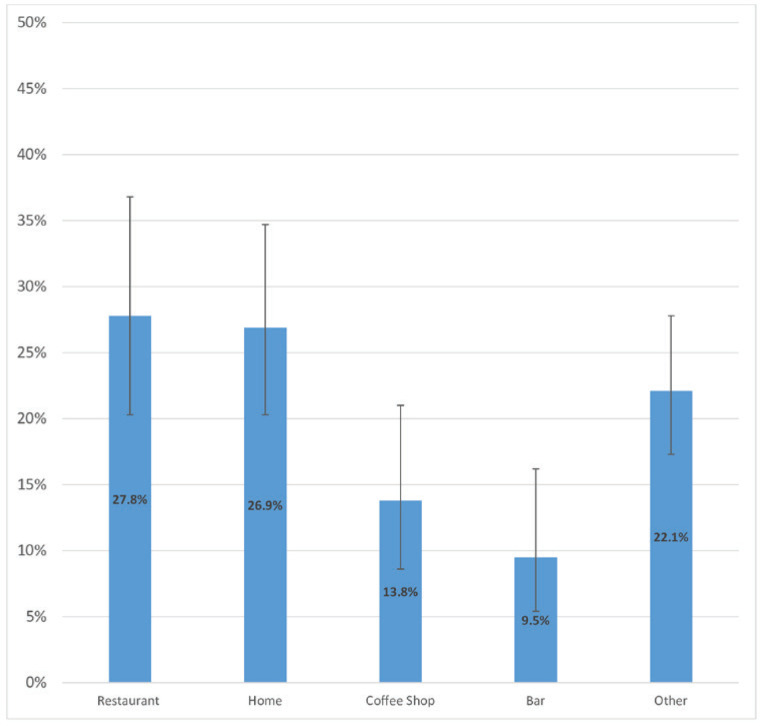
Main shisha smoking venue used by Senegalese students who currently smoke shisha, 2020 Global Youth Tobacco Survey (N=2524)

**Figure 2:**
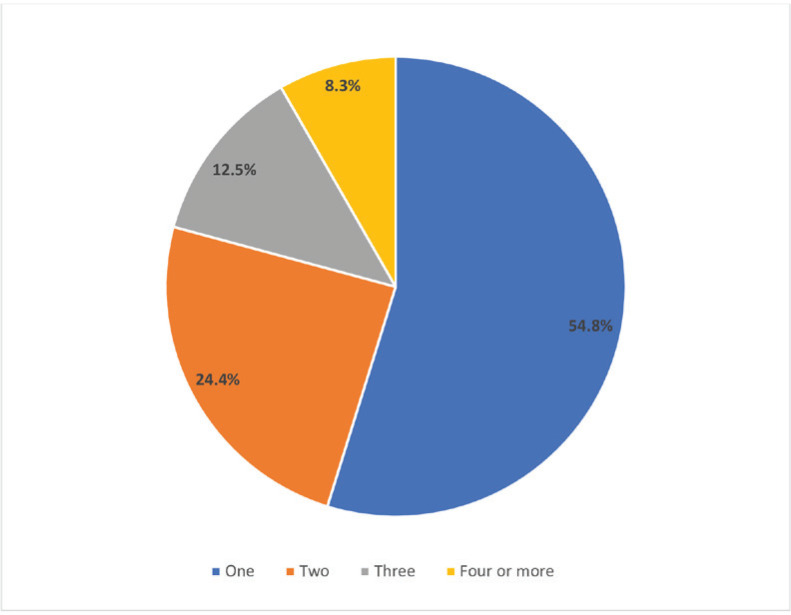
Number of shisha smoking sessions per day reported by Senegalese students who currently smoke shisha, 2020 Global Youth Tobacco Survey (N=2524)

**Figure 3:**
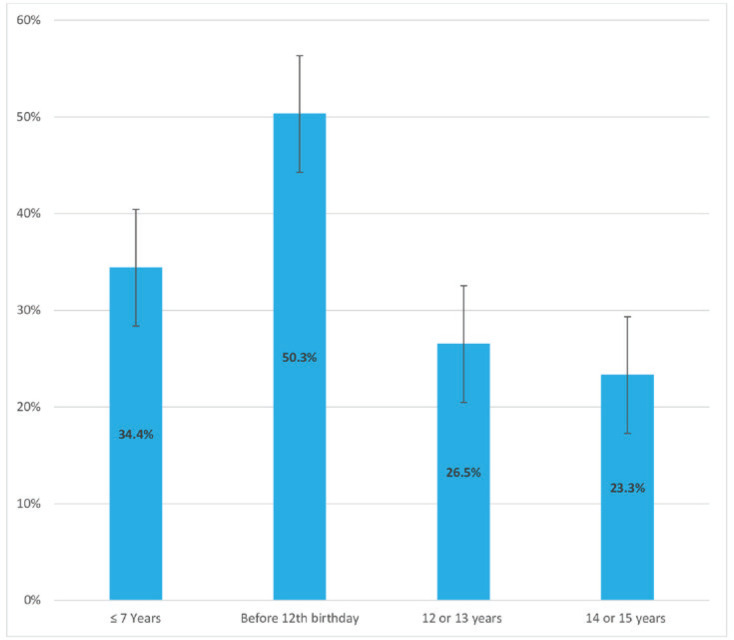
Age at first use of shisha reported by Senegalese students who ever and/or currently smoke shisha, 2020 Global Youth Tobacco Survey (N=2524)

**Figure 4:**
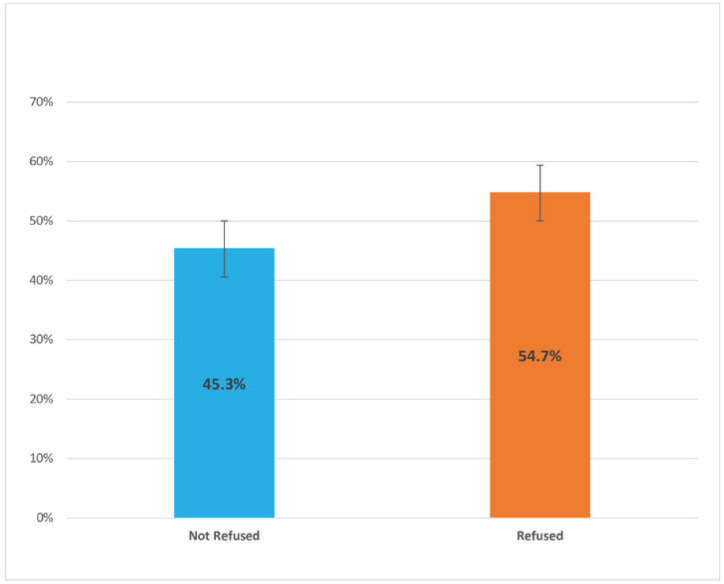
Proportion of Senegalese students who currently smoke shisha that reported someone refused to serve them shisha in the past 30 days because of their age, 2020 Global Youth Tobacco Survey (N=2524)

There were significant differences in the prevalence of both ever and current shisha smoking across different class grades and among students with varying levels of average weekly pocket money. Additionally, the prevalence of both ever and current shisha smoking was significantly higher: 1) among students who currently used smokeless tobacco compared with those who did not, and 2) among students who were exposed to tobacco at home and in public places compared with those who were not. When asked about the venues where they smoked shisha the last time they smoked it, 27.8% (95% CI: 20.3–36.8) of students who currently smoke shisha reported at a restaurant, 26.9% (95% CI: 20.3–34.7) at home, 13.8% (95% CI: 8.6–21.0) at a coffee shop, 9.5% (95% CI: 5.4–16.2) at a bar, and 22.1% (95% CI: 17.3–27.8) other venues (Figure 1). For the number of shisha smoking sessions in a day, 54.8% (95% CI: 42.6–66.3%) indicated one session, 24.4% (95% CI: 15.6–36.2) indicated two, 12.5% (95% CI: 5.8–24.9) indicated three, and 8.3% (95% CI: 3.9–16.7) indicated four or more sessions (Figure 2). When asked at what age they first tried smoking shisha, 34.4% (95% CI: 26.1–43.8) of students who have ever tried and/or currently smoke shisha indicated at the age of ≤7 years. About half (50.3%; 95% CI: 43.2–57.0) of students who ever tried and/or are currently smoking shisha started before their 12th birthday, 26.5% (95% CI: 20.9–33.1) started at age 12 or 13 years, and 23.3% (95% CI:17.3–30.6) started at age 14 or 15 years (Figure 3). More than half (54.7%; 95% CI: 44.7–64.3) of students who currently smoke shisha indicated that someone refused to serve them shisha in the past 30 days because of their age, while 43.3% (95% CI: 35.7–55.3) indicated their age did not deter them from being served shisha (Figure 4).

**Table 2:** The prevalence of ever and current shisha smoking among Senegalese students, Global Youth Tobacco Survey 2020 (N=2524)

*Characteristics*	*Unweighted sample n*	*Ever use of shisha*	*Current use of shisha*
		*% (95% CI)*	*% (95% CI)*
**Sex**			
Girls	1389	6.9 (5.1–9.3)	1.4 (0.9–2.1)
Boys	1111	13.2 (10.4–16.6)	2.9 (1.7–4.8)
p		**<0.001**	**0.021**
**Class grade**			
6 éme (grade 7)	719	8.7 (5.9–12.7)	2.0 (0.9–4.6)
5 éme (grade 8)	1006	9.4 (7.0–12.6)	1.5 (0.8–2.7)
4 éme (grade 9)	549	7.6 (4.7–12.1)	1.0 (0.4–2.1)
3 éme (grade 10)	232	19.7 (10.9–33.0)	7.9 (4.4–13.8)
p		**0.017**	**<0.001**
**Average weekly pocket money** (CFA francs)			
Usually don’t have any money	781	7.1 (5.1–9.8)	1.9 (0.9–4.0)
<2500	1304	9.2 (7.4–11.4)	2.0 (1.3–3.1)
2500–5000	284	14.1 (8.3–23.0)	1.2 (0.4–3.6)
>5000	125	24.3 (13.3–40.1))	8.8 (4.8–15.4)
p		**<0.001**	**0.001**
**Parents’ working status**			
Neither	728	9.9 (5.8–16.5)	2.7 (0.8–8.5)
Father, stepfather, or mother’s partner only	209	8.6 (5.5–13.1)	2.0 (1.0–4.0)
Mother, stepmother, or father’s partner only	1357	10.2 (6.2–16.5)	2.6 (1.1–5.8)
Both	137	10.3 (7.8–13.6)	1.9 (1.3–2.9)
p		0.839	0.870
**Cigarette smoking status**			
Never smoked cigarettes	2180	6.7 (5.0–8.9)	1.0 (0.6–1.5)
Ever tried cigarette smoking but not currently smoking cigarettes	213	25.9 (17.0–37.3)	5.8 (1.9–11.2)
Currently smoking cigarettes	76	50.5 (36.9–64.9)	26.6 (15.4–42.0)
p		**<0.001**	**<0.001**
**Current use of smokeless tobacco**			
No	2337	8.2 (6.3–10.6)	6.8 (2.5–16.7)
Yes	72	38.5 (26.1–52.7)	1.7 (1.1–2.5)
p		**<0.001**	**0.004**
**Exposure to SHS at home**			
No	2069	7.8 (6.0–10.0)	1.3 (0.8–2.1)
Yes	310	22.5 (14.4–33.2)	6.7 (3.6–12.3)
p		**<0.001**	**<0.001**
**Exposure to SHS in public places**			
No	1464	6.7 (5.1–8.7)	1.2 (0.7–2.2)
Yes	974	15.0 (10.8–20.5)	6.7 (3.6–12.3)
p		**<0.001**	**<0.001**
**Parents’ smoking status**			
Neither	1919	8.3 (6.1–11.3)	1.3 (0.7–2.4)
Both	153	12.2 (6.6–21.3)	4.6 (1.8–11.3)
Father only	184	17.8 (12.8–24.3)	6.3 (3.6–10.8)
Mother only	12	16.9 (4.3–47.7)	-
p		**0.008**	**0.002**
**Closest friends’ smoking status**			
None	2192	8.3 (6.5–10.6)	1.4 (1.0–2.2)
Some	177	19.9 (13.9–27.6)	6.5 (3.7–10.9)
Most/all	74	33.8 (20.5–50.4)	13.3 (5.9–27.2)
p		**<0.001**	**<0.001**

CFA Francs: West African Francs (official currency of eight countries in West Africa); 1000 CFA francs about US$1.6. SHS: secondhand smoke.

The factors significantly associated with ever shisha smoking in our fully adjusted model include sex, highest class grade (10th grade compared with 7th grade), average weekly pocket money, parents’ working status (mother only), cigarette smoking status, current use of smokeless tobacco, and exposure to secondhand smoke in public places and at home (Table 3). Boys had twice the adjusted odds of ever smoking shisha compared with girls (AOR=1.97; 95% CI: 1.33–2.92]). Students who had weekly pocket money of >5000 CFA Francs (about US$8)^29^ had three times (AOR=3.29; 95% CI: 1.36–7.95) the adjusted odds to have ever tried shisha smoking compared with those who do not usually have pocket money. Students who had ever tried cigarette smoking but were not currently smoking, and those who were currently smoking cigarettes, had, respectively, 3.69 and 7.54 times the adjusted odds to have ever tried shisha smoking compared with those who had never smoked cigarettes. There were lower odds of ever shisha smoking when the student’s mother/step-mother/father’s partner was the only one working (AOR=0.44; 95% CI: 0.21–0.90) but there was no association when both parents work.

**Table 3:** Factors associated with ever and current shisha smoking among Senegalese students, Global Youth Tobacco Survey 2020 (N=2524)

*Variables*	*Unweighted sample n*	*Ever use of shisha*		*Current use of shisha*	
		*OR (95% CI)*	*AOR (95% CI)*	*OR (95% CI)*	*AOR (95% CI)*
**Sex**					
Girls **®**	1111	1	1	1	1
Boys	1389	2.05 (1.53–2.73) ***	1.97 (1.33–2.92) ***	2.11 (1.11–4.02) *	2.55 (0.94–6.88)
**Class grade**					
6 éme (grade 7) **®**	719	1	1	1	1
5 éme (grade 8)	1006	1.09 (0.63–1.89)	1.21 (0.65–2.26)	0.75 (0.28–2.04)	0.72 (0.23–2.24)
4 éme (grade 9)	549	0.86 (0.44–1.68)	0.84 (0.39–1.82)	0.48 (0.14–1.65)	0.92 (0.22–3.80)
3 éme (grade 10)	232	2.57 (1.26–5.26) **	2.27 (1.10–4.67) *	4.20 (1.58–11.21) **	3.18 (0.96–10.48)
**Average weekly pocket money** (CFA francs)					
Usually don’t have any money **®**	781	1	1	1	1
<2500	1304	1.33 (0.90–1.95)	2.15 (1.22–3.81) **	1.08 (0.48–2.44)	4.76 (1.72–13.22) **
2500–5000	284	2.15 (1.17–3.96) **	2.14 (1.00–4.61) *	0.63 (0.18–2.22)	1.11 (0.22–5.53)
>5000	125	4.20 (2.10–8.42) ***	3.29 (1.36–7.95) **	5.05 (1.91–13.34) **	8.91 (1.75–45.40) **
**Parents’ working status**					
Neither **®**	728	1	1	1	1
Father, stepfather, or mother’s partner only	209	0.85 (0.36–2.00)	0.59 (0.30–1.16)	0.72 (0.18–2.91)	0.71 (0.23–2.24)
Mother, stepmother, or father’s partner only	1357	1.04 (0.47–2.32)	0.44 (0.21–0.90) *	0.95 (0.22–4.16)	0.52 (0.06–4.50)
Both	137	1.05 (0.50–2.19)	0.76 (0.40–1.42)	0.70 (0.17–2.80)	0.38 (0.09–1.51)
**Cigarette smoking status**					
Never smoked cigarettes **®**	2180	1	1	1	1
Ever tried cigarette smoking but not currently smoking cigarettes	213	4.87 (2.88–8.22) ***	3.69 (2.07–6.56) ***	6.22 (2.83–13.71) ***	2.99 (0.93–9.63)
Currently smoking cigarettes	76	14.19 (6.98–28.83) ***	7.54 (2.95–19.29) ***	37.00 (16.28–84.09) ***	21.75 (6.08–77.78)***
**Current use of smokeless tobacco**					
No **®**	2337	1	1	1	1
Yes	72	7.05 (3.72–13.23) ***	11.53 (4.98–26.72) **	4.21 (1.54–11.53) **	8.26 (2.07–33.04) **
**Exposure to SHS at home**					
No **®**	2069	1	1	1	1
Yes	310	3.44 (2.06–5.74) ***	1.62 (0.82–3.22)	5.36 (2.62–10.98) ***	1.40 (0.33–5.86)
**Exposure to SHS in public places**					
No **®**	1464	1	1	1	1
Yes	974	2.46 (1.74–3.49) ***	1.55 (1.00–2.41) *	4.24 (2.05–8.75) ***	1.27 (0.40–4.06)
**Parents’ smoking status**					
Neither **®**	1919	1	1	1	1
Both	153	1.51 (0.68–3.39)	1.10 (0.49–2.46)	3.67 (1.12–12.05) *	1.99 (0.60–6.56)
Father only	184	2.38 (1.50–3.79) ***	1.38 (0.73–2.63)	5.07 (2.46–10.45) ***	3.34 (1.24–8.96) **
Mother only	12	2.23 (0.55–9.15)	1.22 (0.10–2.63)	¶	¶
**Closest friends’ smoking status**					
None **®**	2192	1	1	1	1
Some	177	2.73 (1.80–4.13) ***	1.36 (0.77–2.39)	4.72 (2.55–8.94) ***	1.75 (0.68–4.54)
Most/all	74	5.61 (2.85–11.07) ***	1.84 (0.57–6.02)	10.46 (4.23–25.85) ***	3.94 (0.58–26.91)

AOR: adjusted odds ratio. All the exposure variables (covariates) in the table above were included in the model and variables were mutually adjusted.

® Reference categories.

¶ Not shown because of small numbers. Level of significance of regression results:

* p<0.05,

** p<0.01,

*** p<0.001. CFA Francs: West African Francs (official currency of eight countries in West Africa); 1000 CFA francs about US$1.6. SHS: secondhand smoke.

The factors that were significantly associated with current shisha smoking include higher weekly pocket money, current use of smokeless tobacco, cigarette smoking status, and parents’ smoking status (Table 3). Those who had weekly pocket money of >5000 CFA were 8.91 times as likely to currently smoke shisha compared with students who do not usually have pocket money (AOR=8.91; 95% CI:1.75–45.40). Those who currently smoke cigarettes were 21.75 times as likely to currently smoke shisha compared with those who never smoked cigarettes (AOR=21.75; 95% CI: 6.08–77.78). Father’s cigarette smoking status was also associated with current shisha smoking, compared with students for whom neither parent smoked cigarettes (AOR=3.34; 95% CI: 1.24–8.96). We also conducted gender stratified analysis of prevalence (Supplementary file Table 1) and factors associated (Supplementary file Table 2) with ever and current shisha smoking but the differences were more profound among boys.

## DISCUSSION

Our findings suggest that 1 in 10 students aged 13–15 years has ever smoked shisha and 2.2% currently smoke shisha, despite laws prohibiting the importation, distribution, sale, and use of shisha in Senegal. There are very few similar studies in Sub-Saharan Africa, and it might be difficult to make a meaningful comparison because most of the studies focused on different age cohorts^10,30-33^. The prevalences of both ever and current use of shisha found in our study are higher than those reported in a similar study that used the GYTS 2017 data in Ghana, finding that 3.1% of students aged 13–15 years had ever smoked shisha and the prevalence of current shisha smoking was 1.7%^21^. Another study conducted between 2019 and 2020 in Ethiopia reported ever and current shisha smoking prevalence of 2.6% and 0.6%, respectively, among students aged 14–17 years^10^. We found that the prevalence of shisha smoking was higher among boys compared with girls, in contrast to findings in Ghana where the prevalence among girls was more than double that of boys (2.1% vs. 0.9%) among students aged 13-15 years^21^. However, a study among students aged 12–20 years in The Gambia (which has similar sociocultural characteristics to Senegal), found that ever smoking of shisha was 5.4% among girls and 11.4% among boys^22^. That study did not report the prevalence of current shisha smoking. Based on our findings and the available evidence from other studies^21,22,30^, shisha smoking is a public health problem among students in Sub-Saharan Africa. This is mainly because it is emerging in countries where: 1) it was not previously used, and 2) there is a proliferation of fashionable shisha bars and young people are embracing smoking shisha as social experiment^5^.

Our findings suggest that almost 4 in 10 students in Senegal who currently smoke shisha smoke it in restaurants and bars. Surprisingly, 34.4% of students who have ever tried and/or currently smoke shisha reported they started smoking at age ≤7 years, and 50.3% started smoking shisha before their 12th birthday. Close to half (45.3%) of students who currently smoke shisha indicated their age was not a deterrent from being served shisha. These findings are cause for concern, especially considering shisha smoking outlets, including restaurants and bars, are required by law to deter minors from buying and smoking shisha. Senegal is a signatory to the WHO Framework Convention on Tobacco Control^16^. In addition, it has national laws, including The Tobacco Control Act of 2014, that prohibit the sale of all forms of tobacco products to minors^18^. There is a complete ban on smoking in public places including bars and pubs^17^, as well as the importation, distribution, sale, and smoking of shisha^19^. Therefore, the country has current policies to help protect youth from accessing tobacco, including shisha.

Close to half of students who currently smoked shisha had two or more shisha smoking sessions in a day. Studies have shown that in just a single session of shisha smoking people inhale significantly higher amounts of harmful chemicals and toxic metals compared with smoking a single cigarette^3,11,12,14,34^. Shisha smoking is also associated with low birth weight among infants of mothers who smoke, as well as poor mental health among users^12^. Therefore, preventing youth from accessing shisha might prevent them from the risks associated with shisha smoking.

The factors significantly associated with shisha smoking include cigarette smoking status (ever tried but not currently smoking and current cigarette smoking), current use of smokeless tobacco, exposure to secondhand smoke in public places, getting more pocket money, and having a father who smokes cigarettes. Among students who currently smoke cigarettes, 50.5% have ever tried shisha smoking, and both ever and current use of shisha was strongly associated with the above behaviors. Senegal has made significant efforts in tobacco control including passing regulations that: 1) affirm the public right to health; and 2) regulate the manufacture, packaging, labeling, sale, and use of tobacco products (in 2014)^18^. The importation, sale, distribution, and use of shisha are also banned^19^. Although restaurants and homes were common shisha smoking venues, about 10% of students who currently smoked shisha reported that they last smoked it at a bar. Research has shown that educational and tobacco counter-marketing campaigns are effective in preventing youth from smoking tobacco^35^.

Education about the harms of shisha smoking both in schools and through comprehensive media campaigns that address all tobacco products may be effective for reducing shisha use at home and public places including restaurants. Preventing shisha smoking in public places might reduce exposure of the general public, including youths, to secondhand shisha smoke and prevent initiation of smoking shisha. Considering all the students in the survey were minors (under 18 years), stronger efforts might be implemented to enforce existing age restriction laws in places such as bars and restaurants.

In addition to existing regulations, public health intervention strategies could be effective by focusing on all forms of tobacco use. Prevention and control efforts on shisha smoking might target those with specific risk factors, including those who currently smoke cigarettes, and/or use smokeless tobacco. Future research could also help clarify how policies around importation, distribution, sale, and use of shisha could reduce access to shisha and ultimately reduce the prevalence of shisha smoking among students in Senegal.

### Strengths and limitations

The study was conducted among students; therefore, our findings may not be generalizable to children out of school. Additionally, although we controlled for potential confounders, our study is cross-sectional and there remains the possibility of residual confounding, which limits causal inference.

The main strength of this study is that it is the first study on shisha smoking in Senegal based on nationally representative data of students aged 13–15 years. The study has identified the strongest risk factors associated with shisha smoking, as well as the venues where youths more frequently smoke shisha. This information will be helpful for any public health intervention to curb shisha smoking and tobacco use in general.

## CONCLUSIONS

Our findings suggest shisha smoking is a public health concern in Senegal. School-based education on tobacco use and its consequences, strong tobacco control policies, and comprehensive campaigns that address all tobacco products might prevent initiation and use of shisha. Future research could also help elucidate how policies around importation, distribution, sale, and use of shisha could affect students’ access to shisha – and ultimately reduce the prevalence of use among students in Senegal.

## Supplementary Material



## Data Availability

The data supporting this research are available from the following link: https://www.cdc.gov/tobacco/global/gtss/gtssdata/index.html
