# Supplementary material for: Prevalence and associated factors of shisha smoking among students in Senegal: Global Youth Tobacco Survey 2020
Source: Tob Induc Dis. 2024 May 14;22:10.18332/tid/186656. doi: 10.18332/tid/186656 (PMC11091866; doi:10.18332/tid/186656)
Supplement: Supplementary file 1 [file TID-22-77-s1.pdf]

Supplementary Table 1: The prevalence of ever and current shisha smoking among Senegalese students, 2020 Global Youth Tobacco Survey (n= 2524)

Variable	Boys (n=1111)		Girls (1389)	
	Ever use of shisha	Current use of shisha	Ever use of shisha	Current use of shisha
	% (95% CI)	% (95% CI)	% (95% CI)	% (95% CI)
**Class grade**				
6 éme (grade 7)	12.3(7.2-20.2)	2.8(0.7-10.0)	5.3(3.0-9.4)	1.4(0.8-2.4)
5 éme (grade 8)	12.7(9.2-17.2)	21.(1.0-4.0)	6.7(4.2-10.6)	1.1(0.4-2.7)
4 éme (grade 9)	9.8(5.6-16.7)	1.6(0.7-3.4)	6.0(3.3-10.8)	0.5(0.2-1.8)
3 éme (grade 10)	24.8(12.1-44.2)	9.0(3.7-20.1)	14.9(9.3-23.1)	5.5(2.5-11.9)
	p = 0.125	**p = 0.0373**	**p=0.041**	**p<0.001**
**Average weekly pocket money**				
Usually don't have any money	9.6(6.8-13.2)	2.7(1.1-6.4)	4.5(2.5-8.5)	0.2(0.2-0.3)
Less than 2500 CFA Francs ($4.21) [a]	13.6(10.8-17.0)	2.6(1.4-4.8)	6.1(4.3-8.5)	1.7(1.1-2.5)
2500–5000 CFA ($4.21–8.43) [a]	17.9(9.5-31.3)	2.6(0.8-7.7)	10.6(6.1-17.9)	0
More than 5000 CFA ($8.43) [a]	32.1(13.3-59.2)	9.5(3.3-24.2)	18.7(10.4-31.4)	8.4(3.8-17.5)
	**p = 0.018**	P = 0.130	**p<0.001**	**p<0.001**
**Parent's working status**				
Neither	12.3(5.2-26.5)	3.6(1.2-10.3)	8.2(4.5-14.5)	1.4(0.9-2.2)
Father, stepfather, or mother's partner only	11.7(7.0-18.9)	2.9(1.1-7.1)	5.4(2.9-9.9)	1.2(0.6-2.5)
Mother, stepmother, or father's partner only	10.4(5.8-18.0)	2.6(0.8-8.7)	9.1(4.5-17.4)	1.4(0.4-4.5)
Both	14.8(10.9-19.7)	2.7(1.6-4.5)	7.0(4.8-14.5)	1.4(0.9-2.2)
	p = 0.737	p = 0.648	p = 0.647	p = 0.890
**Cigarette smoking status**				
Never smoked cigarettes	8.5(6.5-11.0)	1.2(0.7-2.1)	5.4(3.7-7.9)	0.8(0.5-1.4)
Ever tried cigarette smoking but not currently smoking cigarettes	30.2(18.0-46.0)	7.1(2.9-16.7)	18.9(11.4-29.7)	4.1(1.2-12.9)
Currently smoking cigarettes	45.9(32.3-60.2)	23.5(12.2-40.5)	72.7(35.3-92.8)	33.9(9.6-71.2)
	**p<0.001**	**p<0.001**	**p<0.001**	**p<0.001**
**Current use of smokeless tobacco**				
No	11.8(9.0-15.3)	2.8(1.7-4.8)	5.2(4.5-8.2)	1.1(0.7-1.8)
Yes	43.3(27.6-60.5)	15.8(6.5-33.7)	35.6(19.0-56.1)	0
	**p<0.001**	**p<0.001**	**p<0.001**	p = 0.5204
**Exposure to SHS at home**				
No	10.4(7.8-13.6)	2.0(1.1-3.6)	5.7(4.2-7.7)	0.8(0.4-1.5)
Yes	30.7(19.4-45.0)	8.0(3.6-16.7)	14.3(7.7-25.1)	4.8(2.0-11.1)
	**p<0.001**	**p = 0.001**	**p = 0.004**	**p = 0.001**
**Exposure to SHS in public places**				

**Variable**	**Boys** (n=1111)		**Girls (1389)**	
	**Ever use of shisha**	**Current use of shisha**	**Ever use of shisha**	**Current use of shisha**
	**% (95% CI)**	**% (95% CI)**	**% (95% CI)**	**% (95% CI)**
No	9.6(7.0-13.1)	2.0(1.0-4.1)	4.4(3.0-6.4)	0.4(0.1-1.2)
Yes	19.3(14.1-25.9)	4.6(2.8-7.6)	11.3(7.1-17.5)	3.2(1.76.0)
	**p = 0.001**	**p = 0.008**	**P=0.002**	**P = 0.002**
**Parent's smoking status**				
Neither	11.63(8.3-16.0)	2.2(1.0-4.7)	5.8(3.9-8.5)	0.6(0.4-1.0)
Both	14.62(6.8-28.5)	3.5(0.9-12.0)	9.9(3.2-26.4)	3.3(0.7-14.6)
Father only	20.5(12.5-31.7)	6.7(2.8-15.0)	13.8(7.5-24.0)	6.2(2.6-14.2)
Mother only	43.6(8.0-87.4)	0	0	0
	p = 0.099	p = 0.163	p=0.183	**p = 0.004**
**Closest friends' smoking status**				
None of them	11.3(8.9-14.1)	2.0(1.1-3.5)	6.2(4.4-8.4)	1.1(0.6-1.8)
Some of them	22.7(14.9-33.0)	8.2(4.7-13.9)	12.6(5.9-25.1)	0.9(0.1-6.7)
Most/all of them	35.0(21.2-51.9)	11.7(3.0-36.5)	33.5(13.2-62.5)	16.3(5.3-40.5)
	**p<0.001**	**p = 0.001**	**p<0.001**	**p<0.001**

CI: confidence interval. OR: unadjusted odds ratio. AOR: adjusted odds ratio. CFA: West African Franc (Official currency of eight countries in West Africa). SHS: secondhand smoke.

Level of significance of regression results: *p<0.05, **p≤ 0.01, *** p≤ 0.001

[a] Using current OANDA Currency Converter. https://www.oanda.com/currency-converter/en/?from=XOF&to=USD&amount=5000

[b] Not shown because of small numbers.

Supplementary Table 2: Factors associated with ever and current shisha smoking among Senegalese students, 2020 Global Youth Tobacco Survey (n= 2524)

	**Boys (n=1111)**		**Girls (1389)**	
	**Ever use of shisha**	**Current use of shisha**	**Ever use of shisha**	**Current use of shisha**
	**AOR (95% CI)**	**AOR (95% CI)**	**AOR (95% CI)**	**AOR (95% CI)**
**Class grade**				
6 éme (grade 7)	Reference	Reference	Reference	Reference
5 éme (grade 8)	1.1(0.5-2.6)	0.6(0.2-2.0)	1.4(0.5-3.8)	1.5(0.1-40.1)
4 éme (grade 9)	0.6(0.2-1.8)	0.5(0.1-2.1)	1.1(0.5-2.6)	5.4(0.3-92.9)
3 éme (grade 10)	2.1(1.0-4.6)	1.4(0.2-8.3)	3.2(1.2-8.7)*	23.2(1.7-324.8)**
**Average weekly pocket money**				
Usually don’t have any money	Reference	Reference	Reference	Reference
Less than 2500 CFA Francs ($4.21) [a]	3.4(1.8-6.2) ***	5.5(1.9-15.7) **	1.1(0.4-3.3)	24.8(0.4-1719.5)
2500–5000 CFA ($4.21–8.43) [a]	2.1(0.9-4.9)	2.6(0.3-20.4)	2.2(0.6-8.6)	[b]
More than 5000 CFA ($8.43) [a]	4.3(1.4-13.6) **	12.6(0.8-197.8)	2.3(0.6-9.8)	125.9(1.2-13217.60*
**Parent’s working status**				
Neither	Reference	Reference	Reference	Reference
Father, stepfather, or mother’s partner only	0.8(0.3-2.5)	0.9(0.2-5.0)	0.6(0.2-1.9)	12.6(0.3-466.3)
Mother, stepmother, or father’s partner only	0.4(0.1-1.3)	[b]	0.5(0.2-1.8)	14.9(0.6-351.5)
Both	1.1(0.4-2.8)	0.6(0.1-4.0)	0.7(0.2-1.8)	3.2(0.4-26.7)
**Cigarette smoking status**				
Never smoked cigarettes	Reference	Reference	Reference	Reference
Ever tried cigarette smoking but not currently smoking cigarettes	3.2(1.8-5.9) ***	3.3(1.3-8.7) *	5.7(1.8-17.8) **	20.5(2.8-147.4) **
Currently smoking cigarettes	6.4(2.5-16. 6) ***	25.1(4.1-155.3) ***	18.9(2.6-138.3) **	228.9(8.3-6309.9) **
**Current use of smokeless tobacco**				
No	Reference	Reference	Reference	Reference
Yes	12.1(4.8-30.8) ***	22.1(4.5-109.7) ***	16.4(4.4-61.5) ***	[b]
**Exposure to SHS at home**				
No	Reference	Reference	Reference	Reference
Yes	1.6(0.7-3.4)	1.3(0.2-7.7)	1.9(0.6-5.7)	7.7(0.8-72.8)
**Exposure to SHS in public places**				
No	Reference	Reference	Reference	Reference
Yes	1.1(0.7-1.9)	0.5(0.1-2.0)	2.4(1.2-4.8)*	13.1(0.5-351.0)
**Parent’s smoking status**				

	Boys (n=1111)		Girls (1389)	
	Ever use of shisha	Current use of shisha	Ever use of shisha	Current use of shisha
	AOR (95% CI)	AOR (95% CI)	AOR (95% CI)	AOR (95% CI)
Neither	Reference	Reference	Reference	Reference
Both	1.2(0.4-3.7)	2.1(0.4-10.2)	0.6(0.2-1.7)	2.3(0.6-8.7)
Father only	1.4(0.6-3.4)	2.9(0.8-10.0)	1.5(0.7-3.4)	14.4(2.1-98.8)
Mother only	2.2(0.1-76.0)	[b]	[b]	[b]
**Closest friends' smoking status**				
None of them	Reference	Reference	Reference	Reference
Some of them	1.9(1.0-3.6) *	3.8(1.2-12.5) *	0.7(0.2-2.0)	[b]
Most/all of them	1.5(0.4-5.6)	5.1(0.4-60.7)	3.0(0.4-22.0)	1.15(0.1-13.1)

N: unweighted sample. CI: confidence interval. OR: unadjusted odds ratio. AOR: adjusted odds ratio. CFA: West African Franc (Official currency of eight countries in West Africa). SHS: secondhand smoke.

Level of significance of regression results: *p<0.05, **p≤ 0.01, *** p≤ 0.001

a Using current OANDA Currency Converter. https://www.oanda.com/currency-converter/en/?from=XOF&to=USD&amount=5000

b Not shown because of small numbers.

All the exposure variables (covariates) in the table above were included in the model and variables were mutually adjusted.

©2024 Cham B. et al.
